# Knowledge and attitude of spouses of puerperas towards breastfeeding

**DOI:** 10.1186/s12905-024-03116-w

**Published:** 2024-05-16

**Authors:** Zhan-Wang Yuan, Li Ma, Yu-Ling Chen, Wen-Li Ge, Hong Zhao, Yun Du, Xiu-Xiu Li

**Affiliations:** 1 Department of Nursing Care (Nursing Department), Northwest Women’s and Children’s Hospital, Xi’an, Shaanxi 710061 China; 2 Department of Nursing Care (Deputy Director of Nursing Department), Xi’an DaXing Hospital, No. 353 of Laodong North Road, Lianhu District, Xi’an, Shaanxi 710018 China; 3 Department of President’s Office, Northwest Women’s and Children’s Hospital, Xi’an, Shaanxi 710061 China; 4 Department of Maternity (Maternity Department), Northwest Women’s and Children’s Hospital, Xi’an, Shaanxi 710061 China; 5 Department of Maternity (Maternity Department), Xi’An QinHuang Hospital, Middle section of Qinhan Avenue, Lintong District, Xi’an, Shaanxi 710061 China

**Keywords:** Breastfeeding knowledge, Infant feeding attitudes, Influencing factors, Puerperas’ spouses

## Abstract

**Objective:**

To investigate the extent of knowledge about breastfeeding and attitudes towards infant feeding among spouses of puerperas at the time of discharge from hospital, and explore the factors influencing spousal attitudes toward breastfeeding.

**Methods:**

We conducted a questionnaire survey among 204 spouses of puerperas who were admitted in the maternity wards at a tertiary hospital in Shaanxi Province between October 2021 and December 2021. Respondents who fulfilled the inclusion criteria were identified using convenient sampling.

**Results:**

(1) The score of breastfeeding knowledge among spouses prior to discharge from the hospital was (10.56 ± 3.78), with an accuracy rate of 59.6%, and the lowest accuracy rate was for Item 1 “Newborns should be fed on time, not on demand” (42.6%) and Item 5 “Breastfeeding can prevent infant rickets” (49.5%). (2) The average score of spouses’ infant feeding attitudes was (58.15 ± 5.55), and the lowest scoring was for Item 17 “Daily urine volume of infants is a reliable indicator to judge whether they get enough breast milk” (1.99 ± 1.14). (3) Generalized linear model analysis showed a more positive attitude (higher score) among spousal attitudes towards infant feeding in those who had received breastfeeding education [OR = 4.588, 95% CI (0.160 ∼ 3.598)] and those with a master’s degree or above [OR = 18.278, 95% CI (3.471 ∼ 9.346)].

**Conclusion:**

(1) Spouses that received breastfeeding education and those that had a Masters Degree and above had more positive attitude towards infant feeding. (2) Medical staff should focus on puerperas’spouses with degrees below master’s level who had not received breastfeeding education. We recommend using a variety of education methods to enable them to acquire more knowledge on breastfeeding and develop a more positive attitude towards breastfeeding, which will further enhance spousal support for breastfeeding, thus positivizing postpartum co-parenting attitudes and improving the rate of exclusive breastfeeding.

## Introduction

Several studies have found that exclusive breastfeeding confers significant benefits and exerts a profound influence on puerperas, newborns, and infants [1–5]. Breast milk is the most natural, safe, and complete food for infants, containing all the nutrients and antibodies essential for their early growth, and is crucial in promoting infant growth and development [6]. The World Health Organization (WHO) recommends that exclusive breastfeeding (EBF) should continue until 6 months, followed by gradual addition of complementary foods until 24 months [7]. The *Action plan for healthy children (2021–2025)* has the goal of achieving an exclusive breastfeeding rate of at least 50% and above for infants aged 0–6 months by 2025 [8]. However, despite the widely recognized benefits of breastfeeding, the current scenario with regard to exclusive breastfeeding is not optimistic. Data from 2014 to 2020 estimate the exclusive breastfeeding rate of infants aged 0–5 months is 44% [9]. The *Investigation Report on Influencing Factors of Breastfeeding in China* published by the China Development Research Foundation found that there are significant regional differences in exclusive breastfeeding rates among big cities, small and medium-sized cities, and rural areas in China, which are 35.6%, 23.3%, and 28.3% respectively, far less than the world average of 44% [10]. The American Academy of Pediatrics [11] (AAP), the American College of Obstetricians and Gynecolo gists (ACOG), Professional organizations such as and the [12] consider breast milk to be the nutritional standard for infant feeding.The World Health Organization (WHO) recommends that exclusive breastfeeding (EBF) should continue until 6 months, followed by gradual addition of complementary foods until 24 months [13]. Infant feeding attitude is an important predictor of infant feeding decisions, which plays a decisive role in compliance with infant feeding guidelines [14, 15], and is also a predictor of breastfeeding duration and exclusivity [16]. A spouse’s attitude towards breastfeeding also influences a mother’s decision to breastfeed and is associated with the initiation and duration of breastfeeding [17]. Therefore, in order to effectively promote breastfeeding, it is necessary to understand the spouse’s attitude towards infant breastfeeding.

The behaviors and attitudes of family members can significantly impact the outlook of puerperas toward breastfeeding throughout the duration of breastfeeding. A systematic study found that despite the differences in the types of breastfeeding activities that spouses participated in, spousal involvement had a positive promotive effect in the initiation and duration of breastfeeding [18]. Supportive attitudes of spouses towards breastfeeding could improve puerperas’ self-efficacy in breastfeeding [19, 20]. Several studies [21–25] reported that spouses engage in the father-infant relationship by participating in breastfeeding activities, such as changing diapers, burping after feeding, bathing, massaging, hugging, singing, or playing with infants. Meanwhile, the spouses also provide emotional and practical support for puerperas in terms of caring for older children, organizing outdoor activities, and so on. Spouses play three types of roles in breastfeeding activities, namely, partnering in decision-making, ensuring the functioning of the family, and offering emotional support for the puerpera. Influenced by the traditional Chinese family culture, most female spouses shoulder the burden of supporting their families, and child-rearing is considered the responsibility of mothers, resulting in a general lack of awareness of breastfeeding among spouses. This lack of correct understanding of breastfeeding results in less spousal participation in breastfeeding, and can bring about feelings of anxiety, helplessness, and abandonment in the parenting process for spouses [26–28]. As important participants and influencers in breastfeeding, spouses ought to change their traditional ways of thinking, proactively acquire knowledge and skills pertaining to breastfeeding, alter their attitudes toward infant feeding, provide more assistance to puerperas, participate in long-term parenting activities, and prolong the duration of breastfeeding.

This study aims to investigate the extent of knowledge about breastfeeding and the attitude towards infant feeding among spouses of puerperas before discharge from hospital and analyzes the factors influencing their attitudes toward breastfeeding. This will help spouses to adopt effective measures to make their attitude more supportive and improve their abilities in breastfeeding activities, and enable them to exert their spousal role better, thereby increasing the breastfeeding rate, prolonging the duration of breastfeeding, and promoting the healthy development of the mother and infant.

## Materials and methods

This was a descriptive study involving 204 spouses of puerperas admitted in the maternity ward of a tertiary hospital in Shanxi province between October 2021 and December 2021. We used convenient sampling and selected spouses who fulfilled the inclusion criteria. We obtained informed consent from puerperas and their spouses prior to the start of the study, and explained in detail the purpose of the study, how to answer the questionnaire, and safeguarding measures we took for the study.

### Inclusion and exclusion criteria

#### Inclusion criteria

Inclusion criteria of puerperas: ≥ 20 years old; singleton pregnancy; gestation ≥ 37 weeks; with no history of psychiatric illness; Apgar scores ≥ 8 at 1 min, 5 min, and 10 min after birth.

Inclusion criteria of spouses: ≥ 22 years old; good language writing and comprehension skills; capable of independently answering the questionnaire; capable of using smartphones; understanding and participating in the study voluntarily.

#### Exclusion criteria

Exclusion criteria of puerperas: Pregnant with abnormal or deformed fetus; with medical conditions that make breastfeeding unsuitable: e.g., HIV infection, active tuberculosis, etc.

Exclusion criteria of spouses: Having a history of mental illness and cognitive impairment; declining to sign the informed consent form.

We determined the clinical sample size (n) according to the quantity of items in the scale, n={[(maximal quantity of items in the scale) × (5–10)] × [1+(10-15%)]}. In this study, the maximal quantity of items was 17, with the sample size n calculated to be = 17 × 10 × (1 + 12%) = 204. The final sample size was 204 cases.

### Research tools

#### General information questionnaire

We compiled and developed a questionnaire based on relevant literature and in consultation with clinical nursing experts. The questionnaire contained details pertaining to spouse’s age, educational background, whether they discussed infant feeding methods with puerperas, the source of breastfeeding knowledge, and so on. We also collected details of maternal education background, pregnancy history, type of delivery, comorbidities during pregnancy and complications, as well as general details of the newborns (gender, bodyweight, body length, and Apgar score).

#### Chinese version of the Iowa Infant Feeding Attitude Scale (IIFAS)

The IIFAS was developed by Mora et al. [29]. , and the Chinese version of the scale was developed by Dai et al. [30] in 2013 with a content validity index (CVI) of 0.99 and Cronbach’s α of 0.635, indicating good reliability and validity. IIFAS assesses two dimensions, namely preference for breastfeeding and preference for formula feeding, and consists of 17 items in total. In this scale, preference for breastfeeding corresponds to eight items, namely, items 3, 7, 9, 10, 12, 13, 15, and 16, while preference for formula feeding corresponds to nine items, namely, items 1, 2, 4, 5, 6, 8, 11, 14, and 17. Each item is scored on a 5-point Likert scale, from 1 (strongly disagree) to 5 (strongly agree). The breastfeeding preference dimension is scored positively, while the formula feeding preference dimension is scored inversely. The total score of the attitude towards infant feeding is the sum of scores of each item, and ranges from 17 to 85. The higher the score, the more positive is the spouse’s attitude towards breastfeeding, and the more inclined is the spouse towards breastfeeding. The total scores can be divided into three grades: ① Positive to breastfeeding (IIFAS scores 70–85); ② Neutral (IIFAS score 49–69); ③ Positive to formula feeding (IIFAS score 17–48) [31]. 

#### Breastfeeding knowledge questionnaire

We measured knowledge of breastfeeding among spouses using a breastfeeding knowledge questionnaire designed by Zhao Min [32]. The questionnaire consists of 17 items pertaining to the benefits of breastfeeding and breastfeeding skills. Each correct answer gets one point, with the total score ranging from 0 to 17. The higher the score, the more is the spousal knowledge on breastfeeding. Content validity index (CVI) of the questionnaire was 0.91, with Cronbach’s α of 0.81–0.86.

### Quality control

#### We conducted the survey before the puerperas were discharged from hospital

Study participants were selected strictly as per the inclusion and exclusion criteria. With informed consent, we provided the link to the questionnaire. The questionnaire was filled out by the spouse and collected back on site.

#### Investigators collected data

When answering the questionnaires, we discouraged the spouses and puerperas from interacting with each other. Any doubts that the respondents had when answering the questionnaires were clarified in a uniform manner.

#### We carefully reviewed all the returned questionnaires

Invalid questionnaires with incomplete data and random responses were deleted. Valid questionnaires were coded.

### Statistical methods

We used SPSS 22.0 software to sort and analyze all the data. Categorical data was described as frequency and percentage. Quantitative data was described using mean ± standard deviation. The variable “attitude towards breastfeeding” was normally distributed as per the normality test analysis. We performed univariate analysis of spouses’ infant feeding attitude using two independent samples t-test and ANOVA. Multivariate analysis of spouses’ infant feeding attitude was conducted using a generalized linear model. *P* < 0.05 was considered statistically significant.

## Results

Between October and December 2021, a total of 254 questionnaires were issued to spouses of puerperas. There were 204 valid questionnaires collected back, with an effective return rate of 80%.

### General information on spouses, puerperas, and infants

Age of spouses ranged from 22 to 43 years, with an average age of (31.61 ± 4.07) years. In terms of occupation, medical staff accounted for 3.9% while non-medical staff accounted for 96.1%, among which 23.5% (48/204 respondents) were company staff and 22.1% (45/204 respondents) were technical staff. Birth weight of newborns was (3.32 ± 0.39) kg and the birth length was (50.23 ± 0.60) cm. (Table 1).

**Table 1:** General information of spouses, puerperas, and infants

Variables		Case (*n*)	Percentage (%)
Age (year)	22∼27	30	14.7
	28∼30	56	27.5
	31∼35	85	41.6
	≧ 36	33	16.2
Ethnicity	Han	198	97.1
	Ethnic minorities	6	2.9
Educational background	Junior college and below	78	38.2
	Bachelor’s degree	96	47.1
	Master’s degree and above	30	14.7
Occupation	Medical staff	8	3.9
	Non-medical staff	196	96.1
Manner of payment of medical expenses	Self-paying	37	18.1
	Medical insurance	167	81.9
Average income per month	< 3000 RMB	13	6.4
	3000–6000 RMB	61	29.9
	6000–10,000 RMB	74	36.3
	> 10,000 RMB	56	27.5
Whether discussed infant feeding methods with parturients	Yes	176	86.3
	No	28	13.7
Spouse’s attitude towards breastfeeding	Pro	190	93.1
	Against	14	6.9
Whether received breastfeeding education	Yes	140	68.6
	No	64	31.4
Puerperas’ educational background	Junior college and below	84	41.2
	Bachelor’s degree	89	43.6
	Master’s degree and above	31	15.2
History of gestation	Unipara	135	66.2
	Multipara	69	33.8
Mode of delivery	Eutocia	95	46.6
	Cesarean	109	53.4
Pregnancy comorbidities or complications	Yes	102	50
	No	102	50
Newborn’s gender	Male	108	52.9
	Female	96	47.1

### Spouses’ knowledge of breastfeeding

The average score of spouses’ knowledge of breastfeeding was (10.56 ± 3.78). Among the 17 items of knowledge on breastfeeding, the 5 items (in ascending order) where respondents had the lowest accuracy were Item 1 “Newborns should be breastfed at specific times instead of on demand,” Item 5 “Breastfeeding can prevent infant rickets,” Item 3 “The more you breastfeed, the more milk you produce,” that is, the amount of milk secretion is related to the frequency of the infant’s feeding, Item 6 “Breastfeeding can prevent diarrhea in the infant,” and Item 9 “Breastfeeding can reduce the incidence of ovarian cancer in mothers.” (Table 2).

**Table 2:** Item-wise ranking of accuracy rate of spouses’ breastfeeding knowledge

Items	Accuracy rate(%)
11. Breastfeeding improves mother-infant bonding.	93.6
14. Breast milk is more nutritious than fresh milk/formula milk for infants.	80.9
8. Breastfeeding can reduce the incidence of breast disease in mothers.	78.9
16. Infants’ jaw should be attached to the breast, and nipple and most of the areola should be contained in infants’ mouth during breastfeeding.	78.4
2. The sooner breastfeeding starts after delivery, the better.	77.5
4. Breastfeeding can avoid childhood obesity.	63.7
7. Breastfeeding can promote the infant’s intellectual development.	63.2
17. Daily urine volume of infants is a reliable indicator to judge whether they get sufficient breast milk.	60.3
12. Breast milk can satisfy all the nutritional needs of infants aged 0–6 months, without additional food or liquids.	59.8
10. Breastfeeding can reduce postpartum bleeding and promote postpartum recovery.	54.9
13. Remaining breast milk should not be discharged after lactation.	54.4
9. Breastfeeding can reduce the risk of ovarian cancer in mothers.	53.4
6. Breastfeeding can prevent infant diarrhea.	52.0
3. The amount of milk secretion is related to the frequency of infant’s feeding, that is, “The more you breastfeed, the more breast milk you produce”.	50.5
5. Breastfeeding can prevent infant rickets.	49.5
1. Newborns should be breastfed at specific times instead of on demand.	42.6

### Sources of knowledge on breastfeeding

**Fig. 1:**
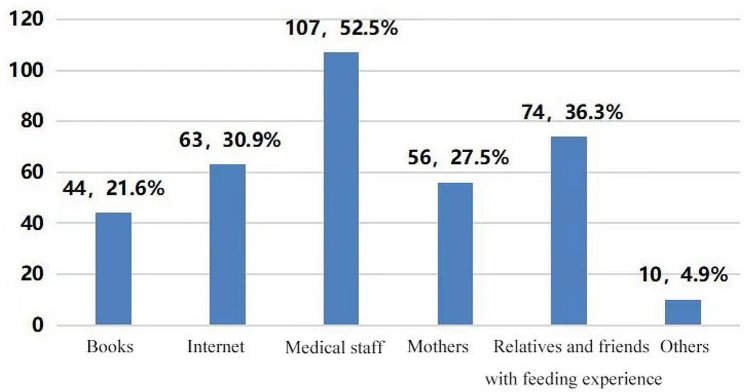
Sources of knowledge about breastfeeding (*n* = 204)

Results showed that the top three sources for spouses to acquire information about breastfeeding were medical staff (107,52.5%), friends and relatives with feeding experience (74,36.3%), and the internet (63,30.9%), while other sources were from mothers, books and magazines, and others. (Fig. 1).

### Spouses’ infant feeding attitude

Ranking of scores of each item of spouses’ infant feeding attitude are shown in Table 3. The average score was (58.15 ± 5.55), among which the average score of preference for breastfeeding dimension was (28.22 ± 4.20) while that of the preference for formula feeding dimension was (29.93 ± 4.29).

**Table 3:** Item-wise ranking of scores of spouses’ IIFAS scores

Items and contents	Score
3. Increase mother-infant closeness.	4.18 ± 0.82
11. Breastfeeding made fathers feel abandoned.	4.10 ± 0.77
9. Breastfeeding is healthier.	4.06 ± 0.90
13. Breast milk is easy to be absorbed.	4.04 ± 0.81
15. Breast milk is convenient.	3.71 ± 0.81
4. Breast milk is iron-deficient.	3.59 ± 0.80
14. Formula milk is healthier.	3.55 ± 0.75
5. Infants fed formula milk eat more.	3.50 ± 0.86
8. It is not advisable to breastfeed in public.	3.49 ± 1.04
2. Formula milk is more convenient.	3.35 ± 0.91
1. Benefits of breastfeeding are interrupted when discontinued.	3.24 ± 1.00
10. Infants fed by breast milk eat more.	3.17 ± 0.85
16. Breast milk is inexpensive.	3.16 ± 1.12
6. Formula milk feeding makes it easier for mothers to resume their work.	3.12 ± 0.96
12. Breast milk is a complete food.	3.06 ± 0.97
7. Formula milk feeding deprives infants of maternal love.	2.84 ± 1.05
17. Occasional drinking is not suitable for breastfeeding.	1.99 ± 1.14

### Univariate analysis of spouses’ infant feeding attitude

Breastfeeding is a volatile behavior easily affected by a variety of factors. In this study, we carried out a univariate analysis of eight aspects including spouses’ age, educational background, whether they received breastfeeding education, and mother’s pregnancy history, as well as type of delivery. The homogeneity test of variance showed equal total variance of IIFAS in each group (*P* > 0.05). Univariate analysis showed statistically significant differences between the groups in education background and whether the spouse had received breastfeeding education (*P* < 0.05). There was no significant difference in the total score of IIFAS among other groups (*P* > 0.05). (Table 4).

**Table 4:** Univariate analysis of general information on spouses and puerperas (*n* = 204)

Items	Case (*n*)	Score of IIFA (‾x ± s)	t/F value	*P* value
Age (year)			0.489	0.690
22∼27	30	57.10 ± 4.63		
28∼30	56	58.57 ± 4.77		
31∼35	85	58.29 ± 5.75		
≧ 36	33	58.00 ± 6.97		
Educational background			11.196	0.000
Junior college and below	78	56.86 ± 4.88		
Bachelor’s degree	96	57.93 ± 4.68		
Master’s degree and above	30	62.20 ± 7.70		
Occupation			1.686	0.093
Medical staff	8	61.38 ± 9.02		
Non-medical staff	196	58.02 ± 5.36		
Average income per month			0.533	0.660
< 3000 RMB	13	57.54 ± 6.58		
3000–6000 RMB	61	57.72 ± 5.79		
6000–10,000 RMB	74	58.03 ± 4.82		
> 10,000 RMB	56	58.91 ± 5.99		
Whether discussed infant feeding method with parturients			0.553	0.581
Yes	176	58.23 ± 5.49		
No	28	57.61 ± 5.97		
Whether received breastfeeding education			2.631	0.009
Yes	140	58.83 ± 5.79		
No	64	56.66 ± 4.68		
History of gestation			-0.635	0.526
Unipara	135	57.97 ± 5.19		
Multipara	69	58.49 ± 6.21		
Mode of delivery			-0.631	0.529
Eutocia	95	57.88 ± 5.46		
Cesarean	109	58.38 ± 5.65		

### Multivariate analysis of spouses’ infant feeding attitude

In this study, collected data were continuous variables and normally distributed. IIFAS score of spouses was taken as the dependent variable, spouses’ age, education background, average income per month, whether the spouse discussed infant feeding methods with puerperas, whether the spouse received breastfeeding education, puerperas’ education background, and pregnancy comorbidities and complications were taken as the independent variables. Assignment of independent variables are shown in Table 5. A generalized linear model (GLM) was used to analyze differences in the distribution of total scores of feeding attitude between groups. Results showed that the IIFAS score was higher in spouses who received breastfeeding education (OR = 4.588, 95% CI (0.160 ∼ 3.598)). Spouses with education background of master’s degree or above obtained higher IIFAS scores (OR = 18.278, 95% CI (3.471 ∼ 9.346)). (Table 6) .

**Table 5:** Value assignment of independent variable in the generalized linear model

Variable	Items	Value assignment
X1	Age (year)	22–27 = 1, 28–30 = 2, 31–35 = 3, ≧36 = 4
X2	Educational background	Junior college and below = 1, Bachelor’s degree = 2, Master’s degree and above = 3
X3	Average income per month (RMB)	< 3000 = 1, 3000–6000 = 2, 6000–10,000 = 3, > 10,000 = 4
X4	Whether discussed infant feeding method with parturients	Yes = 1, No = 2
X5	Whether received breastfeeding education	Yes = 1, No = 2
X6	Parturient’s education background	Junior college and below = 1, Bachelor’s degree = 2, Master’s degree and above = 3
X7	Pregnancy comorbidities or complications	Yes = 1, No = 2

**Table 6:** Multivariate analysis of IIFAS scores (*n* = 204)

Group	β	95% CI		OR	*P*
		Lower limit	Upper limit		
[Educational background = 1]	0^a^				
[Educational background = 2]	1.218	-0.632	3.069	1.665	0.197
[Educational background = 3]	6.408	3.471	9.346	18.278	< 0.001
[Whether received breastfeeding education = 1]	1.879	0.160	3.598	4.588	0.032
[Whether received breastfeeding education = 2] Constant term	0^a^ 26.221^b^	. 21.595	. 31.837	.	.

## Discussion

### Status and sources of knowledge on breastfeeding

In our study, we identified that spouses had limited knowledge of breastfeeding based on the five items of breastfeeding knowledge with the lowest accuracy rate, and among these, there were three items (Item no. 5, 6, and 9) pertaining to the benefits of breastfeeding for mothers and infants. The lowest accuracy rate of only 42.6% was for Item 1 “Newborns should be fed on time, not on demand,” indicating that most spouses were not aware of the new concept of on-demand breastfeeding. On-demand breastfeeding is crucial in newborn feeding as it is key to ensuring the quality and quantity of breast milk. It can not only drain the mother’s breasts in time, but also promote the regeneration of breast milk, enabling mother and infant to quickly adjust to each other, ensuring full feeding of the infant and promoting the infant’s growth and development. Therefore, we recommend that the importance of on-demand breastfeeding should be emphasized in the information and education that is given to spouses. Zhao et al. [32] found that the accuracy rate of Item 17 “Daily urine volume of infants is a reliable indicator to judge whether they get enough breast milk” was the lowest (50.7%), while the accuracy rate of this item in our study was higher (60.3%). This may be related to the fact that obstetric nurses ask the spouses of puerperas to monitor and record the urination and defecation of newborns every day and use this to observe the status of the infant’s feeding. In this study, we found that spouses’ knowledge of breastfeeding mainly came from medical staff, relatives and friends with feeding experience, and the internet. These findings are consistent with the interview results of Joseph et al. [33] among rural mothers in northwestern Nigeria. Medical staff remain the best source of information on infant feeding during antenatal hospitalization, suggesting that medical staff play an important role in communicating information pertaining to breastfeeding. However, our findings are different from the study by Zhao [32], which showed that the main source of knowledge was books (99/146, 67.81%). The findings in our study may be related to the fact that puerperas’ spouses were in frequent contact with medical staff right from the time of pregnancy to the postpartum period, and this gave the spouses more convenient access to obtain knowledge of breastfeeding. Moreover, most spouses were willing to seek help from medical staff when they encountered problems due to their trust in the professional medical staff.

### Spouses’ attitude towards infant feeding

In this study, the average total score of IIFAS in spouses was (58.15 ± 5.55), which belonged to the moderate level. Han et al. [34] investigated the IIFAS scores of 215 spouses in a hospital in Taiwan during their stay (59.60 ± 6.93). A study by lkay et al. [35] on 200 spouses in Istanbul University Hospital in Turkey showed IIFAS score (60.65 ± 6.69). The average score of IIFAS for males in Nigerian [36] communities was (58.60 ± 7.60). The results of this study are basically consistent with the above research results.

In Nigeria [36] the first two items of the Community male survey were 16,3; The score below 3 was 6.8.17. Han et al. [34] investigated the first two items of single score as 3,13; Below 3 points (1, 2, 6, 17). In this study, the first two items of single score were 3 and 11; The two items alone are below 3 points (7, 17). In the above studies, item 3 scored at the highest single level, that is, breastfeeding increased mother-child intimacy, indicating that both mothers and spouses recognized the benefits of breastfeeding for mother-child relationship. Shukri et al. [37] conducted a survey on 88 primiparas 2 weeks postpartum, and found that the scores of mothers in the exclusive breastfeeding group and the non-exclusive breastfeeding group were 67.0 ± 6 and 64.4 ± 5, respectively, showing no statistical difference, both higher than the results of this study, which was related to the different subjects of the study. Some scholars believe that the total score of IIFAS between 57.7 and 60.0 is “attitude neutral” [38]. Therefore, in this study, spouses have a relatively positive attitude towards infant feeding, which is related to the fact that most of the respondents have a higher education level, work and live in first-tier cities, the rapid development of Internet information, and more ways to receive knowledge about breastfeeding in daily life. They have a good fertility awareness and a positive attitude towards breastfeeding. Medical personnel in hospitals and communities should provide information on knowledge and skills related to breastfeeding to mothers’ spouses during pregnancy and after childbirth, and encourage their spouses to participate in breastfeeding activities throughout the whole process, especially the spouses of new fathers, so that they can adapt to and change roles more smoothly.

### Analysis of factors influencing spouses’ infant feeding attitudes

Current literature in China has plenty of research on maternal feeding attitudes but few on that of spouses. Spouses’ support for breastfeeding not only improves the proportion of puerperas willing to breastfeed after delivery, but also prolongs the duration of breastfeeding [39]. In our study, univariate analysis showed that spouses with a master’s degree or above and those who had received breastfeeding education had higher infant feeding attitude scores and were more inclined to exclusive breastfeeding. This was consistent with the findings of Wang [40] suggesting that the higher the education background, the more positive the attitude towards breastfeeding. Regina et al. [41] conducted a questionnaire survey on 151 spouses in the maternity ward of a public hospital in Singapore. In their subgroup analysis, spouses with higher education (bachelor’s degree or above) (no statistical difference between groups) who attended antenatal classes were found to acquire more knowledge of breastfeeding and therefore support breastfeeding activities. Educational background being related to cognitive ability, the higher the education background of the spouse, the more information on breastfeeding they acquired, and this helped them develop a scientific feeding attitude. In addition, there was a significant positive relationship between breastfeeding education and feeding attitudes, and the spouses who had received breastfeeding education had more positive attitudes toward breastfeeding.

## Conclusion and prospects

This study demonstrated that spouses had a neutral attitude towards breastfeeding, but they did not fully understand the benefits of breastfeeding for the mother and infant and had insufficient knowledge about breastfeeding. In the baseline survey, 31.4% of spouses in the outpatient department did not receive breastfeeding education, indicating that the coverage of breastfeeding education during pregnancy needs to be improved. We recommend improving access to such information though “one-on-one” guidance in the midwife clinic, outpatient video education, brochures, and so on. In addition, nursing staff should focus on spouses with an education background below master’s degree and who have never received breastfeeding education. Promoting breastfeeding through “Internet+” technology to disseminate knowledge and skills will enable them to be more effective in their role as parents by changing the spouse’s attitude towards breastfeeding and improving their ability to offer support. This will help puerperas to develop self-confidence in breastfeeding and eventually achieve the goal of extending the duration of breastfeeding.

### Limitations of the study

This study only investigated puerperas’ spouses before discharge from hospital. We did not include municipal and district (county) level hospitals, and hence our limited findings could not highlight any differences between regions. In addition, we did not longitudinally track the status of knowledge about breastfeeding and infant feeding attitudes after discharge in this study. We recommend evaluating the extent of spouses’ involvement in breastfeeding in future studies to help in offering targeted measures.

## Data Availability

All data generated or analysed during this study are included in this article. Further enquiries can be directed to the corresponding author.
