# Correction: Knowledge and attitude of spouses of puerperas towards breastfeeding

**DOI:** 10.1186/s12905-024-03252-3

**Published:** 2024-07-17

**Authors:** Zhan-Wang Yuan, Li Ma, Yu-Ling Chen, Wen-Li Ge, Hong Zhao, Yun Du, Xiu-Xiu Li

**Affiliations:** 1 Department of Nursing Care (Nursing Department), Northwest Women’s and Children’s Hospital, Xi’an, Shaanxi 710061 China; 2 Department of Nursing Care (Deputy Director of Nursing Department), Xi’an DaXing Hospital, No. 353 of Laodong North Road, Lianhu District, Xi’an, Shaanxi 710018 China; 3 Department of President’s Office, Northwest Women’s and Children’s Hospital, Xi’an, Shaanxi 710061 China; 4 Department of Maternity (Maternity Department), Northwest Women’s and Children’s Hospital, Xi’an, Shaanxi 710061 China; 5 Department of Maternity (Maternity Department), Xi’An QinHuang Hospital, Middle section of Qinhan Avenue, Lintong District, Xi’an, Shaanxi 710061 China

**Correction: BMC Women’s Health (2024) 24:289**

10.1186/s12905-024-03116-w

Following publication of the original article [1], the author noticed couple of errors and typos in the published version,

The authors observed that in-text reference citations lack ending punctuation. To ensure proper citation formatting, end periods to the end of each in-text citation added (“Zhao et al. [32].” in Status and sources of knowledge on breastfeeding, “Han et al. [34]. and lkay et al. [35].” in Spouses’ attitude towards infant feeding under the Discussion section).

For clarity the author would like to add a sentence “The results of this study are basically consistent with the above research results” after (58.60 ± 7.60) and change the word “puerariums” to “primiparas” in Spouses’ attitude towards infant feeding under the Discussion section.

In reference list, the reference 21 should read as “DeMontigny F, Gervais C, Larivière-Bastien D, St-Arneault K. The role of fathers during breastfeeding. Midwifery. 2018;58:6–12. 10.1016/j.midw.2017.12.001” instead of “deMontigny F, Gervais C, Larivière-Bastien D, St-Arneault K. The role of fathers during breastfeeding. Midwifery. 2018;58:6–12. 10.1016/j.midw.2017.12.001”.

The original article has been corrected.
